# 3D Camera and Single-Point Laser Sensor Integration for Apple Localization in Spindle-Type Orchard Systems

**DOI:** 10.3390/s24123753

**Published:** 2024-06-09

**Authors:** R. M. Rasika D. Abeyrathna, Victor Massaki Nakaguchi, Zifu Liu, Rizky Mulya Sampurno, Tofael Ahamed

**Affiliations:** 1 Graduate School of Science and Technology, University of Tsukuba, 1-1-1 Tennodai, Tsukuba 305-8577, Japan; s2136030@u.tsukuba.ac.jp (R.M.R.D.A.); s2330270@u.tsukuba.ac.jp (V.M.N.); s2230267@u.tsukuba.ac.jp (Z.L.); s2236019@u.tsukuba.ac.jp (R.M.S.); 2 Department of Agricultural Engineering, University of Peradeniya, Kandy 20400, Sri Lanka; 3 Department of Agricultural and Biosystem Engineering, Universitas Padjadjaran, Sumedang 45363, Indonesia; 4 Institute of Life and Environmental Science, University of Tsukuba, 1-1-1 Tennodai, Tsukuba 305-8577, Japan

**Keywords:** integrated sensor system, apple localization, robotic apple harvesting

## Abstract

Accurate localization of apples is the key factor that determines a successful harvesting cycle in the automation of apple harvesting for unmanned operations. In this regard, accurate depth sensing or positional information of apples is required for harvesting apples based on robotic systems, which is challenging in outdoor environments because of uneven light variations when using 3D cameras for the localization of apples. Therefore, this research attempted to overcome the effect of light variations for the 3D cameras during outdoor apple harvesting operations. Thus, integrated single-point laser sensors for the localization of apples using a state-of-the-art model, the EfficientDet object detection algorithm with an mAP@0.5 of 0.775 were used in this study. In the experiments, a RealSense D455f RGB-D camera was integrated with a single-point laser ranging sensor utilized to obtain precise apple localization coordinates for implementation in a harvesting robot. The single-point laser range sensor was attached to two servo motors capable of moving the center position of the detected apples based on the detection ID generated by the DeepSORT (online real-time tracking) algorithm. The experiments were conducted under indoor and outdoor conditions in a spindle-type apple orchard artificial architecture by mounting the combined sensor system behind a four-wheel tractor. The localization coordinates were compared between the RGB-D camera depth values and the combined sensor system under different light conditions. The results show that the root-mean-square error (RMSE) values of the RGB-D camera depth and integrated sensor mechanism varied from 3.91 to 8.36 cm and from 1.62 to 2.13 cm under 476~600 lx to 1023~1100 × 100 lx light conditions, respectively. The integrated sensor system can be used for an apple harvesting robotic manipulator with a positional accuracy of ±2 cm, except for some apples that were occluded due to leaves and branches. Further research will be carried out using changes in the position of the integrated system for recognition of the affected apples for harvesting operations.

## 1. Introduction

The recent development of sensors and electronics has improved the quality and accessibility of robotic applications in agriculture, including apple harvesting [1], estimation of fruit yield [2], growth monitoring [3], and autonomous navigation. In the last few decades, several applications of robotic apple harvesting have undergone significant developments and made significant contributions. The integration of sensors to improve the accuracy of robotic apple harvesting has significantly improved over the past few years. However, most combined sensor fusion systems and sensor systems involve complex approaches for calibration and applications, and it is challenging to achieve complete harvesting success [4,5,6].

The robotic apple harvesting system has two major components: the vision system and the robotic arm, which is called a manipulator. The end of the manipulator links to an end effector to harvest apples from trees [7]. The end effector is responsible for picking up or detaching the target apple once the manipulator brings the end effector near the target apple in the tree. The key feature for most successful attempts to reach the target apple is accurate coordinates provided by the vision system [8]. Most large-scale and medium-scale orchard farmers attempt to mechanize their farming activities due to the high demand for production and low-skilled labor [9,10]. Thus, most fruit orchard operations are carried out by manual laborers with low efficiency, and there is a high demand for skilled laborers during the harvesting season. In terms of ergonomic injuries, quality, and quantity of harvesting, more attention has been given to replacing human labor with robotics, especially in orchard apple fruit production.

Apple harvesting requires large numbers of the seasonal labor force, and the failure of timely harvesting can cause enormous production losses. With the demanding manual labor trends, orchard farmers must adapt developing technologies to their daily orchard operations [11]. Apple harvesting robots consist of manipulators, vision systems, control systems, and autonomous or manual vehicles for carrying the system inside the orchard. Vision systems play a major role in the detection of apples based on feature extraction with the help of state-of-the-art detection networks [12], followed by obtaining and calculating coordinates to detect apples and sending those coordinates to the manipulator in real time. The manipulator follows the calculated trajectory path based on vision systems to position the gripper and finally grab the target apple.

**Figure 1:**
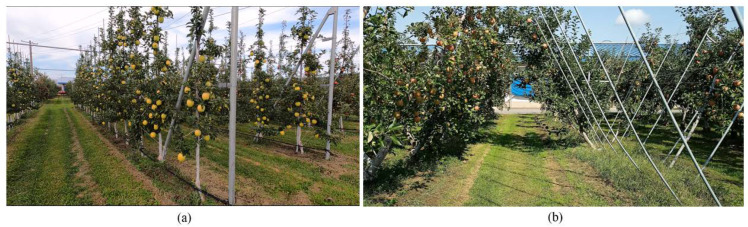
(**a**) Tall spindle apple orchard architecture system. (**b**) V-shaped apple orchard architecture system at the Aomori Prefectural Apple Research Institute in Kuroishi, Aomori Prefecture, Japan.

Robotic arm apple picking requires at least three coordinates, which include X, Y, and Z (the depth value). Vision sensors can be used in real time to obtain these coordinates. Several studies have been conducted in which robotic arms operate based on a 3D camera that provides X, Y, and Z coordinates, followed by a combination of sensors to improve the accuracy. Moreover, the localization of apples in the novel developed orchard training systems, such as spindle wall types, tall spindles, and V/Y-shaped systems, enables more success due to the nature of the trained trees, which have more open areas for apples (Figure 1). Thus, genetic improvements led to the use of single apples instead of apple clusters.

However, researchers have attempted to use 3D cameras along with robotic systems under outdoor conditions, either in real orchard or simulated orchard conditions [13,14]. The main drawback of outside operation is the variation of light, followed by shadows and dark spots. In indoor conditions where light variations are minimal, robotic apple picking results in high accuracy, whereas outdoor experiments show less accuracy. One of the main reasons for this is that the vision system cannot provide accurate depth values or distance information for the robotic system to reach the target apples.

The challenge to improve dynamic harvesting by robotic systems is to incorporate an accurate vision system that can provide precise depth localization coordinates even if the robotic arms are in a stationary state, which is the future trend of harvesting robots. The recently developed RealSense D455f camera is able to provide an accurate RGB frame with the help of an infrared filter. Laser range finders, which use the principle of time of flight (ToF), can provide depth values with high precision even under varying outdoor light conditions and over long distances. Combining the RGB frame with laser depth values from a laser range finder can improve the accuracy of apple localization coordinates. Thus, the objective of this study was to develop a sensing system integrated with an Intel^®^ RealSense™ Depth Camera D455f and a single-point laser ranger (PLS-K-100, 635–645 nm red laser) to obtain accurate localization coordinates under different outdoor illumination conditions.

## 2. Related Work

### 2.1. RGB-D Cameras for Apple Localization for Harvesting Systems

Fruit recognition and localization are the key features of robotic harvesting systems. Over the years, different sensors have been used for the visualization of fruits [15]. At the beginning of detecting apples for robotic applications, fruit color texture and shape were used as basic features to distinguish them from other objects [16]. Object detection networks have been used for different fields of applications [17,18] and with different training systems to improve the accuracy of the models based on the applications [19]. Improving the conventional detection models to perform faster [20] is challenging, which is essential, especially for agricultural applications.

Different convolutional neural network (CNN)-based architectures, such as YOLOv3 [21], YOLOv5 [22], YOLOv7 [23], Faster RCNN [24], Mask RCNN [9], EfficientDet [25], and CenterNet, which have been trained based on apple datasets, have been used for detection and localization with high accuracy. Initially, 2D cameras were used as color sensors [26,27] to identify the apples, and the 2D information provided faced interference resulting from variations in light conditions. A study was conducted with a multiclass detection model to localize apples for robotic harvesting, which included branch/wire, leaf, fruit, and non-occluded classes. An attempt was made to avoid obstacles and reduce damage to the apples [28].

The advancement of the use of nonvisible wavelengths created a way to develop multispectral, hyperspectral [29], and thermal [30,31] cameras for fruits based on different parameters. Accurate localization requires 3D information about the environment. To obtain 3D cloud information, LiDAR (light detection and ranging) [32] has been used based on the time-of-flight principle (ToF) [33]. Stereo matching is the key principle of binocular vision. A study was carried out to locate apple branch obstacles based on stereo vision, and the results showed that the error was 6.20 mm [34]. Apple orchard point cloud processing based on LiDAR has achieved successful results, and experimental results have shown 85% detection success in Fuji apples [6] because these fruits cause greater light backscattering than leaves and trunks. A study has been conducted to localize red and bicolored apples and to cluster the apples based on the random sample consensus (RANSAC) algorithm; the authors used a light shield to reduce direct sunlight [35].

Robotic approaches for harvesting apples require accurate depth values [36] from the center point of the gripper to the detected apples. Stereo depth cameras calculate the depth of each pixel based on stereo disparity with the help of the triangulation principle. Stereo depth camera target localization depends on the level of correspondence matching, image quality, repetitive scenes, and illumination variations [37]. The cameras that project lights with known patterns are based on an emitter called a structural-light depth camera, and the depth information is obtained by comparing the original pattern of the light and the deformed pattern obtained from the receiver. Thus, varying illumination conditions in agricultural environments limit the accuracy of stereo depth cameras and structural light cameras [38].

The active RealSense cameras consist of three lenses, an RGB camera, an IR camera, and an IR laser projecting source. The stereo vision principle overlaps the left- and right-side sensors and calculates depth values [39]. This process is supported by an IR laser projector capable of sending IR patterns and detecting reflections to generate depth frames for each pixel [40,41]. An RGB-D camera that combines RGB and depth framers can be used as a single sensor to map the environment in three dimensions. A study was conducted based on a 3D camera and Mask RCNN, with a three-degree-of-freedom manipulator for harvesting apples based on a vacuum suction end effector [14]. The information obtained from RGB-D cameras with the help of deep neural networks (DNNs) plays a significant role. The detection and localization results were obtained from the RGB-D sensors used for different applications in apple orchard systems.

Intel^®^ RealSense™ D435 [1] and D455™ (Intel Corporation, Santa Clara, CA, USA) [25] cameras were used to localize the apples, and the grasping pose was estimated based on the processing point cloud obtained from depth streams [42], but the results showed average accuracies of 0.61 cm and 4.80° degrees from the center position and orientation, respectively. The previous study [25] that we conducted was based on the state of art (SOTA) of detection algorithms: YOLOv4, YOLOv5, YOLOv7, and EfficientDet combined with a RealSense D455 camera to measure the accuracy of apple detection in terms of depth values at the dynamic stage. According to the results, we found that EfficientDet outperforms with higher accuracy than other networks as regards other detection models, compared with the RMSE values. Most of the depth scanning errors were observed due to variations of light and shadows from leaves and branches. Intense sunlight and changes in camera scanning create errors, so combining depth sensors that can provide accurate depth values under varying light conditions with RGB-D cameras could provide more precise localization values for robotic apple applications.

### 2.2. Integrated Sensor Systems for Apple Harvesting

Integrating two or more sensors can overcome the limitations of obtaining accurate depth values in robotic apple harvesting. The depth values and positional information are the key features in the vision system because the vision system receives considerable noise due to variations in light conditions.

Another study was conducted by combining LiDAR and a 3D camera. After obtaining accurate extrinsic metrics between the LiDAR and the camera, the LiDAR was used for sensing the geometric information, and the RealSense D455 was used for detecting apples. The results showed that for highly accurate depth values, the standard deviations of 0.5, 1.2, and 1.8 m were 0.253, 0.230, and 0.285 cm, respectively [43]. Furthermore, an active laser scanning scheme (ALACS) was introduced with the help of an FLIR camera to improve localization accuracy, and laser scanning was used to recalculate the 3D position of the apple by comparing the depth frame obtained from the RealSense RGB-D camera. Robust calibration was conducted based on the random sample consensus method for calibrating the model parameters related to collected data [44,45]. The sensor fusion method that has already been used requires complex calibration methods, especially when fusion was conducted with LiDAR [43] or high processing power based on the complexity of the algorithm, which requires high computational power in applications with robotic systems.

The variation of light intensities and shadows from the leaves cause errors when obtaining the apple localization coordinates using 3D cameras, especially the depth values (z-axis coordinates), which leads to inaccurate robotic apple harvesting operations. This study was carried out by integrating a RealSense™ D455f camera and a single-point laser range sensor to avoid the effect of light variations when obtaining the depth values. The single-point laser was attached to two precision servo motors for locating the center position of the detected apple. Compared with the sensor fusion method, this sensor-integrated approach required less computational power and easy calibration. A new system is proposed to obtain apple localization coordinates in an outdoor environment under static conditions to enable harvesting operations from tree to tree. Static conditions are considered for the harvesting of individual trees to avoid damage. This paper presents the following contributions to the development of integrated sensors for apple harvesting:

- The RealSense D455f color frame was integrated with a single-point laser range sensor as a new low-cost high-accuracy integrated depth sensing system to determine the values for obtaining accurate localization coordinates of apples in a spindle-type orchard for the development of a robotic arm.
- The 3D camera integrated with a single-point laser ranger at different levels of light conditions was used to increase the accuracy of the localization of apples and to analyze the ability of the 3D camera to overcome the illumination effect during the day.

## 3. Materials and Methods

### 3.1. Data Preparation for Apple Detection

Accurate detection of apples is the most important role of robotic actuators. Based on our previous study, we found that the EfficientDet object detection algorithm outperformed dynamic depth measurements [25]. However, we found that even though the D455 RealSense camera with the EfficientDet network could provide accurate depth values when changing the light variations, there were many limitations for obtaining constant accurate depth values for robotic apple harvesting. This study was carried out to link an Intel^®^ RealSense™ Depth Camera D455f and a single-point laser (PLS-K-100 laser ranging module (PAIOUJIDIAN, Shanghai, China)) with a light source wavelength of 635~645 nm, a laser spot size of 10 m and 5 mm, a response time of ≥0.3 s, and an anti-ambient light of 300 klx) to obtain more precise localization results under different light conditions.

**Figure 2:**
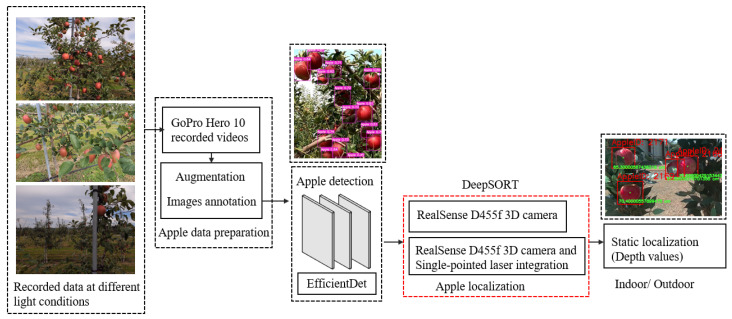
Data preparation and overall workflow for the development of an integrated sensing system using a 3D camera and a single-point laser.

We used the EfficientDet-based apple detection model from our previous study [25]. The mean average precision (mAP@0.5) was 0.775, and the model was trained using dataset images collected from the Aomori Prefectural Apple Research Center, Research Institute in Kuroishi, Aomori Prefecture, Japan, using a GoPro Hero 10 (GoPro, Inc., Woodman Labs, Inc., San Mateo, CA, USA). The dataset was collected under different light conditions throughout the day (Figure 2).

### 3.2. Development of an Integrated Sensor System

**Figure 3:**
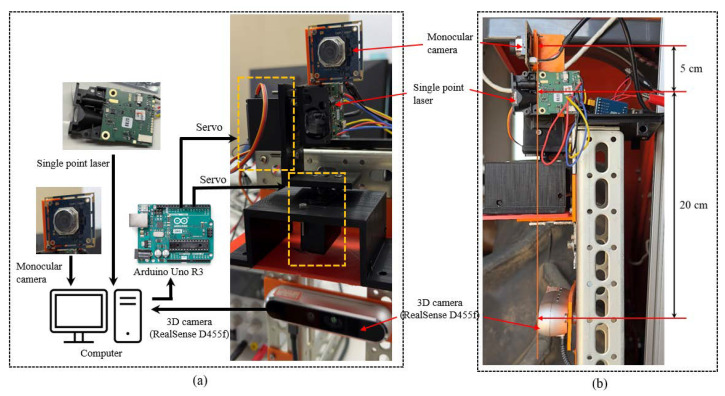
Developed integrated sensing system: (**a**) connection diagram with the computer (**b**) mounted behind the tractor, side view.

The RealSense camera depth values were compared with the depth values of the integrated sensor system (RealSense camera + single point laser). The single-pointed laser sensor was mounted on two servo motors (ICQUANZX MG995 Analog Servo Metal Gear Servo 20 KG high-speed torque digital servo motor) with a monocular camera (ELP USB Camera Module Autofocus 100 degree no-distortion lens, full HD, Shenzhen Ailipu Technology Co., Ltd., Shenzhen, China) that was used to track the apples and head the laser. The servo motors were attached to an Arduino Uno^®^ to control the movement angles of the integrated unit (Figure 3).

**Figure 4:**
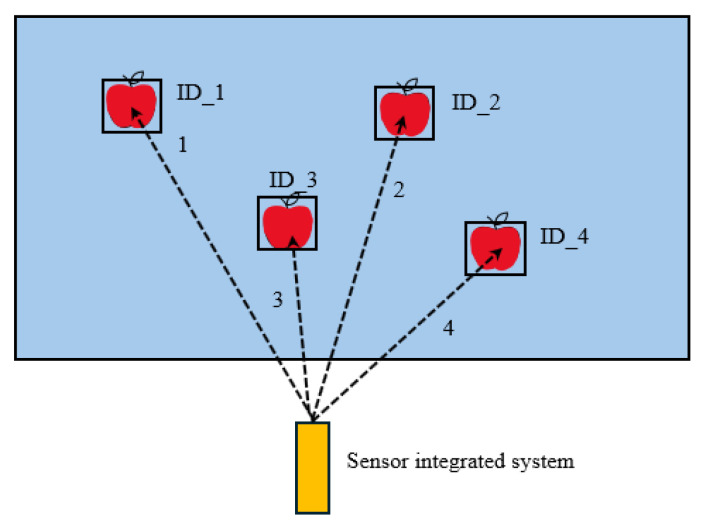
Principles of single-pointed laser ranger operations for the detection of apples based on apple IDs, the number 1 to 4 indicate the moving sequence of single point laser.

The distance between the 3D camera and the single-point laser was 20 cm in height and 5 cm in the lateral direction between the laser and monocular camera. Compared to other D400 cameras, the RealSense D455f 3D camera was upgraded with a 750 nm near-infrared (NIR) filter, which improved the depth measurements by avoiding false detections caused by light leakage. The key concept of the integrated sensor system was to move the single-point laser ranger to the center of the detected apples, and the moving sequence was arranged based on the detection ID generated by the DeepSORT algorithm implemented in our previous study (Figure 4).

### 3.3. Calibration of the 3D Camera and Laser Range Finder

**Figure 5:**
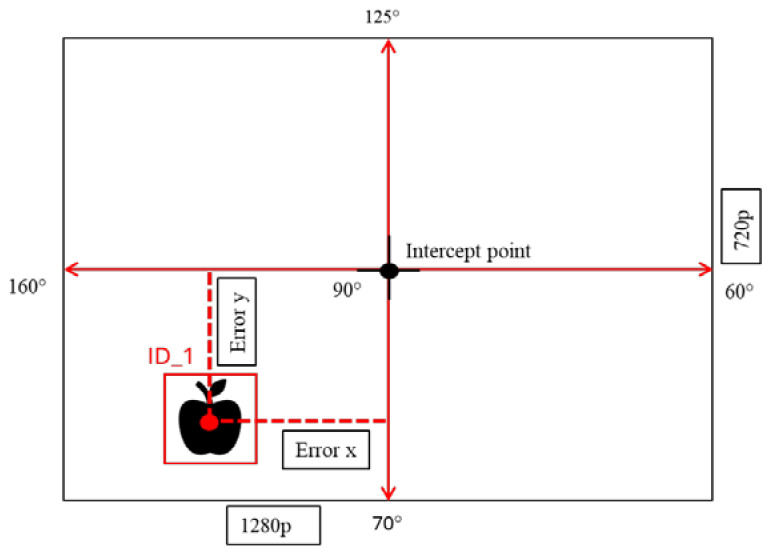
Principle of servo motor operation to track the detected apple from the Apple ID.

**Figure 6:**
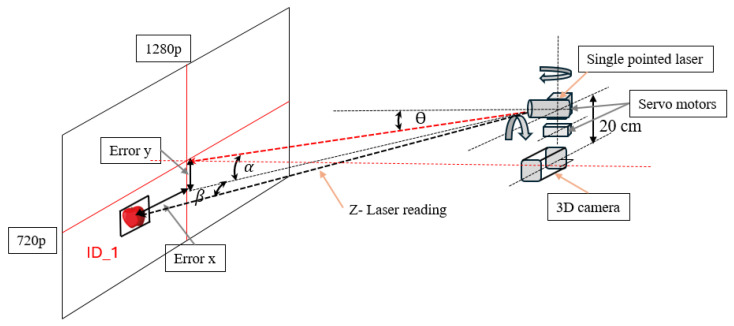
Apple position and picking coordinates.

The integrated sensing system was calibrated to obtain the depth values based on calculating the angles for each center position of detected apples (Figure 5) in relation to the center point of the image frame as an interception of point positions. The angle calculations for the servo motors are explained in Equations (1) and (2).

(1) $$Servo\_x=\alpha \left[x_{i}\right]+\left(x-x_{i}\right)\times \frac{\alpha \left[x_{i+1}\right]-\alpha \left[x_{i}\right]}{x_{i+1}-x_{i}}$$

(2) $$Servo\_y=\beta \left[y_{i}\right]+\left(y-y_{i}\right)\times \frac{\beta \left[y_{i+1}\right]-\beta \left[y_{i}\right]}{y_{i+1}-y_{i}}$$

where x or *y* are the center coordinates of a detected apple, $x_{i}$ or $y_{i}$ is the lower value of frame size (720), $x_{i+1}$ or $y_{i+1}$ is the minimum frame resolution (1280), $\alpha \left[x_{i}\right]$ and $\beta \left[y_{i}\right]$ are the maximum servo angles, and $\alpha \left[x_{i+1}\right]$ and $\beta \left[y_{i+1}\right]$ are the minimum servo angles, respectively. In the camera frame indicated in Figure 5, the servo motors were calibrated based on pixel values. The horizontal moving servo motor (Servo_x) covered 1280 pixels by moving 60° degrees to 160° from left to right. The vertical servo (Servo_y) covered 720 pixels by moving 70° to 125° degrees. The single-point laser was 20 cm above the 3D camera and the laser point was directed to the middle of the camera frame at the beginning (Figure 6). The vertical servo moved θ (15° degrees) downward to align with the canter position of the camera frame.

Based on Figure 6, the apple localization coordinates were obtained, and the obtained coordinates were converted into robotic arm moving coordinates depending on the calibration process between the vision systems and the robotic arm parameters.

The integrated sensor system and a computer (11th Gen Intel^®^ Core™ i7-11700F@2.50 GHz, Nvidia^®^ GeForce RTX™ 3060 (12 GB GPU; 16 CPUs), Santa Clara, CA, USA, and 16 GB RAM with Windows^®^ 10 home edition™) were installed on the on-board system of a four-wheel tractor for forward movement, and the system was evaluated in an artificial orchard architecture to compare the depth values of the 3D camera and integrated sensor system in a static state for tree-to-tree operation. The system was powered using a power inverter (MWXNE Sine Wave, 12 V, 100 V, 1200 W, Max 2400 W) connected to the tractor battery.

### 3.4. Setup of Indoor and Outdoor Experiments for Obtaining Static Depth Values

Indoor and outdoor experiments were conducted to evaluate the integrated sensing system with 3D camera depth values at Tsukuba Plant Innovation Research Center (T-PIRC), University of Tsukuba (36°7′9.5304″ N, 140°5′44.5518″ E). The indoor experiment was conducted under light conditions of 476~600 lx. The artificial spindle apple orchard architecture was created indoors, the apples were placed at 30 different locations in the canopy, and depth values were obtained.

Usually, apples that are occluded by branches and leaves are difficult to harvest via robotic system; instead, in this study, we focused on the visible apples or apples that were partially occluded by the leaves, which have the greatest potential for accurate robotic harvesting from a static state. In a previous study [25], we showed that variations in light and wind conditions created false depth values in dynamic detection, even under static conditions once the depth values were obtained apart from the false depth readings; at times, the RealSense D455 and D455f cameras with different detection networks achieved depth values of zero. These sudden changes in depth cannot be used for smooth robotic apple harvesting systems.

An outdoor experiment was conducted under different light conditions to analyze the developed integrated sensor system to obtain more precise depth values for robotic applications. The experiment was conducted at different times that also included variable cloud conditions and variations in lighting in the morning and afternoon. The light values were measured using a digital light meter (SMART SENSOR, Digital lux meter AS803, accuracy ±5% rdg ± 10, measurement range 1~200,000 lux, Wanchuang Electronic Prod. Co., Ltd., Dongguan, China), and the outdoor data were collected under light conditions of 1963~2000 × 10 lx, 3470~3600 × 10 lx, 4519~4700 × 10 lx, 7710~7900 × 10 lx, 8800~8900 × 10 lx, and 1023~1100 × 100 lx. For each light condition, 30 apple locations were used to obtain the distance information, which was compared with the 3D camera depth values or equivalent distance information. The true measurement depth for each apple location was measured using a laser range finder (minimum distance of 0.2 m, maximum range of 200 m, ±5 mm accuracy, Leica^®^ Disto™ classic 5; Hexagon AB, Leica Geosystems Holdings AG, St. Gallen, Switzerland).

**Figure 7:**
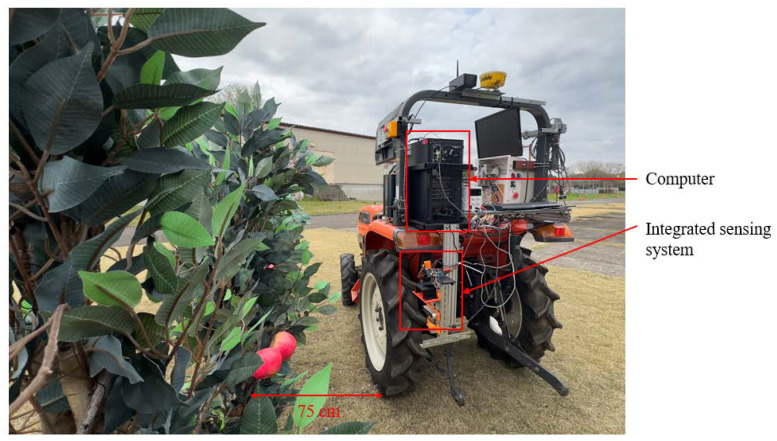
Outdoor experimental setup for obtaining static depth values of apples under different light conditions in an artificial spindle orchard.

The integrated sensor system was mounted on a four-wheel tractor, and the outdoor conditions were evaluated. The system was evaluated at a static condition; when obtaining the localization coordinates, the vision system was not moved, and after obtaining the localization coordinates from one tree, the tractor was moved to another tree parallel to the tree raw for obtaining the coordinates of the next tree. The spindle orchard architecture was arranged based on artificial trees. The integrated sensor system was kept 75 cm away from the artificial tree row, assuming that the 75 cm distance from the tree canopy could be easily accessible for robotic manipulators for apple harvesting (Figure 7).

The RMSE (root-mean-square error) values were calculated (Equation (3)) by comparing the real depth values obtained from the laser range finder, the RealSense 3D camera, and the integrated sensor system.

(3) $$\;RMSE=\sqrt{\sum_{i=1}^{N}\frac{{\left(Z_{i}-{Z’}_{i}\right)}^{2}}{N}}$$

where *i* is the observation value at *N* number of objects, $Z_{i}$ refers to the depth values from a 3D camera or integrated system, and ${Z’}_{i}$ is the ground reference or true depth values. The result, RMSE, provides an overall measure of the prediction errors, with lower values indicating more accurate predictions.

For each light condition, the laser range finder readings were checked with measured tape values to determine whether the laser range finder affected the true distance information. We measured the ground reference depth values two times for each apple location since the 3D camera and servo-mounted laser were 20 cm apart from each other.

## 4. Results

The training results of the YOLOv4, YOLOv5, YOLOv7, and EfficientDet detection system are listed in Table 1. The IDs of the detected apples were obtained via the DeepSORT tracking algorithm. The integrated sensor system followed the ID values to move the single-pointed laser to obtain the depth values of the apples. These data were collected from our previous research, and based on the RMSE values for the depth measurements, EfficientDet showed fewer error values compared to other models. Moreover, for this study we used the EfficientDet detection model to evaluate the performance of the developed integrated sensing system.

### 4.1. Indoor Experimental Results

**Figure 8:**
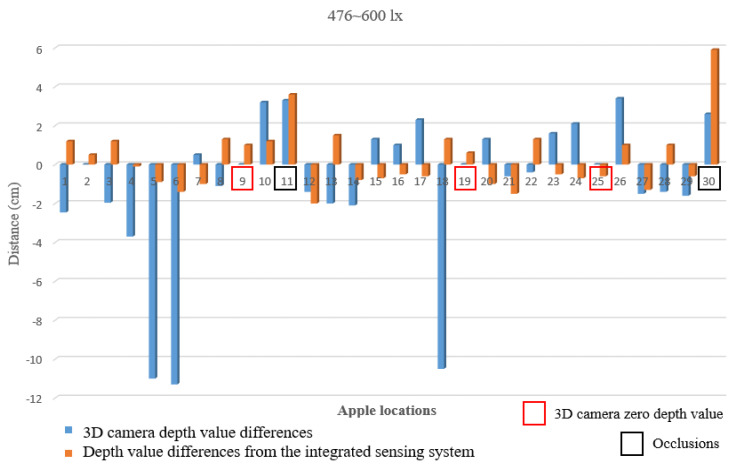
Differences in the depths of the indoor experimental setup (476~600 lx).

Most of the robotic systems tested under indoor conditions, especially without varying light conditions, perform well; however, when tested outside under the varying conditions of vision systems, they fail to provide accurate localization coordinates. The RMSE value of the indoor experiment was 1.62 cm, which included the values of apples that were covered with leaves, and the differences in the depth values from the real depth values are illustrated in Figure 8.

The apples at locations 9, 19, and 25 were detected by the 3D camera; however, the depth values were 0 cm. The integrated sensing system was able to obtain accurate depth values. At locations 11 and 30, apples were observed while occluded by the leaves.

### 4.2. Results of the Outdoor Experiment

**Figure 9:**
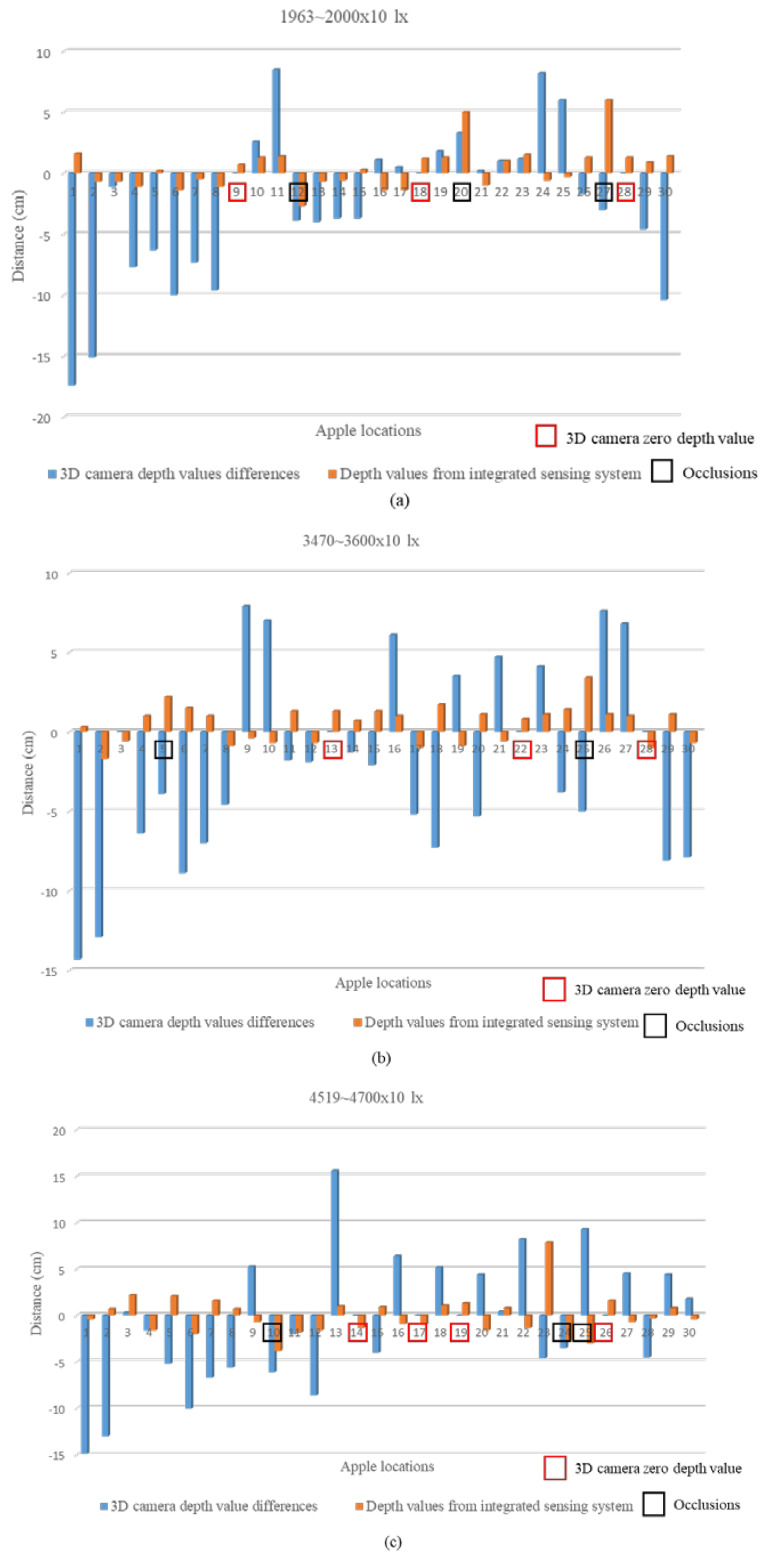

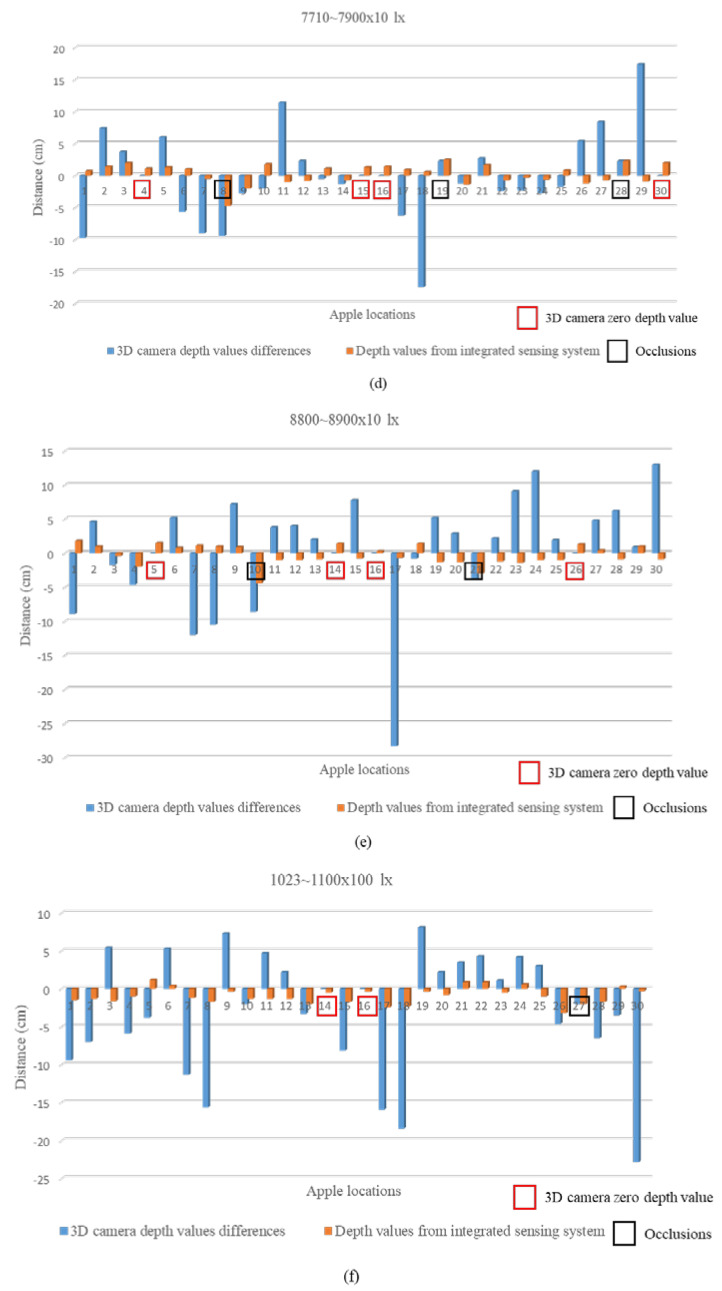
The differences in depth during the outdoor experiment under different light conditions: (**a**) 1963~2000 × 10 lx (10 a.m. JST, cloudy day), (**b**) 3470~3600 × 10 lx (11 a.m. JST, cloudy day), (**c**) 4519~4700 × 10 lx (3 p.m. JST, cloudy day), (**d**) 7710~7900 × 10 lx (10 a.m. JST, sunny day), (**e**) 8800~8900 × 10 lx (11:30 a.m. JST, sunny day), and (**f**) 1023~1100 × 100 lx (1:30 p.m. JST, sunny day).

Outdoor experiments were conducted under different light conditions to compare the differences between the 3D camera values and the integrated sensing detection results. Figure 9 shows the resulting depth values.

The apple locations that were detected by the 3D camera and given zero depth values are highlighted in red, the apples that were occluded are highlighted in black, and the occlusion was due to leaves and branches (Figure 9). In outdoor conditions, the wind also moved the leaves and occluded the apples with leaves at the point of measurement.

According to the results, the integrated sensor system showed a minimum and maximum error of ±2 cm, except for the values for occluded apples, which is applicable for accurate robotic apple harvesting applications under outdoor conditions. Compared with the RMSE values under different light conditions, the 3D camera RMSE values increased with increasing light intensity (Table 2).

The RMSE values were calculated, including the depth values of occluded apples, based on the results from the integrated sensing system, which was able to perform well, with a maximum error of 2.13 cm, which showed at the light intensity 4519~4700 × 10 lx (3 p.m., JST, cloudy day). The 3D camera RMSE values showed high deviations of errors when the light intensity values were increased because of this problem. The robotic system combined with the 3D camera had problems, showing different performances during different light conditions and leading to failures.

## 5. Discussion

Apple harvesting robotic systems became popular after apple orchard architecture changed to a simple canopy structure in which the robotic systems could easily reach the apples. The spindle, tall spindle, and Y/V-shaped apple orchard architectures helped to develop unique robotic systems, such as parallel arm robotic systems, to increase the efficiency of harvesting operations. Even though simple apple tree architectures exist, occlusions and environmental effects, such as variations in light conditions, hinder the performance of vision systems. Most robotic applications use the RGB-D camera, where detection models utilize the RGB frame to identify the locations of the detected apples with the camera frame and depth frames to utilize the distance to detect apples. The integration of accurate depth sensing of a single laser ranger with a RealSense RGB-D camera provides highly accurate depth values, which can be used for robotic apple harvesting applications.

### 5.1. Deep Learning-Based EfficientDet Detection Network

In harvesting applications, the apples that are occluded with branches, supporting cables, and leaves are difficult to localize with vision systems, and those apples are difficult to harvest with robotic systems. In most cases, the vision systems are mounted on the robotic arm or the robotic system vehicle and pointed directly to scan the canopy only from one direction. The apples that are occluded can be reached if the vision system is capable of scanning the same apples at different angles and directions [46]

The EfficientDet detection network was selected as the detection network since EfficientDet was capable of providing highly accurate localization results in a dynamic state in our previous study. In this study, we sought to develop an integrated sensor system that can reduce localization errors for more precise robotic applications.

**Figure 10:**
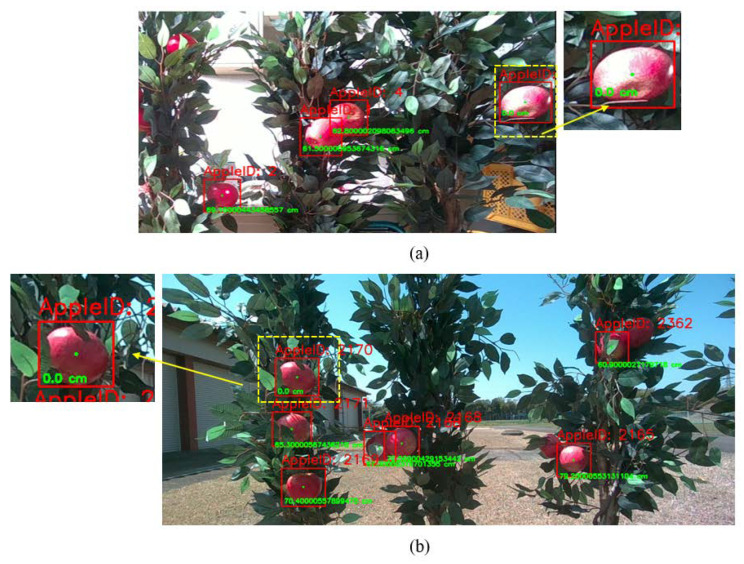
Three-dimensional camera depth values: (**a**) indoor example of a zero-depth value for a localized apple and (**b**) outdoor example of a false depth value.

Moreover, the depth frame accuracy of the RealSense D455f camera changed due to light variations, shadows, and leaf occlusion. There were some instances in which the detection network detected the apples, and the depth values were indicated as zero. This could be due to several reasons: one reason could be the variation of lighting, another could be the reflection of light from each apple, and occlusions resulting from wind and shadows could also be valid reasons. Moreover, the wind could slightly change the location of the apples, which was also a reason why some of the 3D camera depth values became zero. As indicated in Figure 10, a 3D camera sometimes results in 0 cm depth values under indoor and outdoor conditions, which needs to be avoided for accurate robotic manipulation.

### 5.2. Integrated Sensing System

In this study, our focus was to develop a vision system that could accurately localize the apples that were fully visible or partially occluded by the leaves, since we tried to scan the canopy only from one direction. As indicated in the methodology, the integrated sensor system was kept 75 cm away from the apple trees since the RealSense D455f camera could provide depth values starting from 60 cm. The single-pointed laser range finder was mounted on two servo motors, which helped to point the laser to any position of the camera frame, as calculated based on camera pixel coordinates.

**Figure 11:**
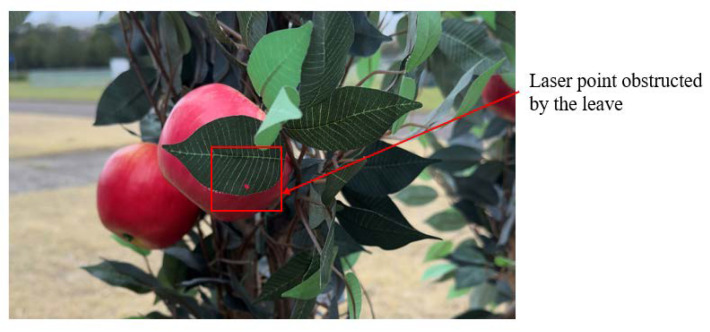
The single-pointed laser obstructed by a leaf.

There were some occasions when the laser range finder was unable to reach the apples accurately. As indicated in Figure 11, the laser beam was obstructed by the leaves; to avoid this kind of error, the vision system should have been capable of analyzing the same target at different angles or obtaining several depth values in the detected area to identify the occlusions and analyze the variations.

Moreover, the apples that were covered by branches and poles failed to obtain accurate localization results. The laser pointed only to the center positions of the apples detected by the EfficientDet detection network. Thus, we found that the integrated system and 3D camera localization coordinates were more accurate when the target was near the center of the camera frame because of the laser beam divergence angle. If robotic systems can only focus on harvesting apples detected in the center of the camera frame, the accuracy of harvesting can increase. Accurate geometrics of the target locations help the robotic systems to achieve proper grasping sequences while avoiding misleading robotic cycles and obstacles.

### 5.3. Application Environment

Researchers have attempted to develop parallel robotic arm systems for apple harvesting based on developed orchard architectures such as spindle, high spindle, and X/Y. These architectures provide more space and accessibility to robotic systems to reach the target apples than do conventional apple orchard systems. Our study was conducted based on spindle-type orchard architecture, and we used an artificial orchard structure outdoors to analyze the developed sensing system.

The performance of the integrated sensing system was evaluated under static conditions. This system can be combined with different robotic systems, single robotic arms [47], and parallel robotic arm systems [48] to improve the harvesting accuracy; thus, this approach can be extended to dynamic localization for faster robotic applications. One of the limitations of this system was the obstructions from the leaves, branches, and metal poles, which generated false depth values. Another limitation is the limited availability of spindle orchards: most of the new spindle orchards have started to be prepared and require at least 3 to 4 years to harvest. This research focused on future perspectives regarding the wide use of a low-cost high-accuracy integrated sensing system for spindle orchards and the automation and development of robotic apple harvesters.

The proposed system had advantages compared with complex calibration systems as well as economic perspectives. The developed system had a single laser and two servo motors coupled with a 3D camera, which is required to calculate the servo angles and point the laser to detected apples in sequence. The 3D LiDAR and 2D LiDAR are expensive and difficult to link with the 3D camera. Moreover, processing large numbers of data coming from sensing systems required high-end computational power and slowed down the follow-up robotic applications as well. This developed integrated sensing system can overcome the single sensor 3D camera limitations in robotic applications.

## 6. Conclusions

Precise apple localization coordinates are required for robotic apple systems to deploy accurate harvesting cycles. The localization results from 3D cameras were affected by the variation in light conditions, which can be avoided by the integration of a single-pointed laser ranger with the 3D camera. This study was conducted by integrating a RealSense D455f camera and a PLS-K-100 laser ranging module, and the following conclusions can be drawn as a new contribution from this research:

1. The EfficientDet deep learning-based detection network mAP@0.5 of 0.775 was capable of accurately detecting apples under different light conditions with a RealSense D455f camera from spindle-type orchard datasets.
2. The developed integrated sensing system, combined with a RealSense D455f 3D camera and a single-pointed laser ranger mounted on two servo motors, could provide accurate depth values of ±2 cm compared to 3D camera positional information.
3. The integrated sensing system was used under different light conditions. In the spindle-type orchard conditions, the RMSE values of the RGB-D camera depth values and integrated sensing systems varied from 3.91 to 8.36 cm and from 1.26 to 2.13 cm, respectively, at different times of day and under different environmental conditions.
4. The developed low-cost high-accuracy integrated systems can be incorporated with robotic systems to localize apples under outdoor static conditions for harvesting apples at tree locations in spindle-type orchards.
5. The apple localization coordinates (X, Y, and Z) values can be obtained from the proposed integrated system, and the coordinates transferring to the robotic arm can be done based on the calibration process between the robotic arm and the vision system.

Further research will be carried out to incorporate the new low-cost and high-accuracy integrated sensing system with the robotic arm for harvesting apples in spindle-type orchard conditions.

## Figures and Tables

**Table 1:** The training results of YOLOv4, YOLOv5, YOLOv7, and EfficientDet apple detector models.

	mAP@0.5	Precision	Recall
EfficientDet [25]	0.775	0.950	0.950
YOLOv4 [25]	0.840	0.840	0.790
YOLOv5 [25]	0.861	0.874	0.783
YOLOv7 [25]	0.905	0.892	0.828

**Table 2:** The RMSE values of the 3D camera and integrated sensor system under different light conditions.

	Indoor	Outdoor					
Light intensity (lx)	476~600	1963~2000 ×10 (10 a.m., JST, cloudy day)	3470~3600 ×10 (11 a.m., JST, cloudy day)	4519~4700 ×10 (3 p.m., JST, cloudy day)	7710~7900 ×10 (10 a.m., JST, sunny day)	8800~8900 ×10 (11:30 a.m., JST, sunny day)	1023~1100 ×100 (1:30 p.m., JST, sunny day)
3D camera (cm)	3.91	6.52	6.24	6.67	6.66	8.05	8.36
Integrated sensing system (cm)	1.62	1.82	1.26	2.13	1.56	1.46	1.39

## Data Availability

The dataset that was generated and analyzed during this study is available from the corresponding author upon reasonable request, but restrictions apply to the data reproducibility and commercially confident details.
